# Glomerular Immune Deposits Are Predictive of Poor Long-Term Outcome in Patients with Adult Biopsy-Proven Minimal Change Disease: A Cohort Study in Korea

**DOI:** 10.1371/journal.pone.0147387

**Published:** 2016-01-22

**Authors:** Sung Woo Lee, Mi-Yeon YU, Seon Ha Baek, Shin-Young Ahn, Sejoong Kim, Ki Young Na, Dong-Wan Chae, Ho Jun Chin

**Affiliations:** 1 Department of Immunology, Seoul National University Postgraduate School, Seoul, Korea; 2 Division of Nephrology, Department of Internal Medicine, Seoul National University Bundang Hospital, Seongnam, Korea; 3 Department of Internal Medicine, Seoul National University College of Medicine, Seoul, Korea; Emory University, UNITED STATES

## Abstract

**Background and Objectives:**

There has been little published information on risk factors for poor long-term outcome in adult biopsy-proven minimal change disease (MCD).

**Methods:**

Data from sixty-three adult, biopsy-proven primary MCD patients treated at a tertiary university hospital between 2003 and 2013 were analyzed. Baseline clinical and pathologic factors were assessed for the associations with composite outcome of creatinine doubling, end stage renal disease, or all-cause mortality.

**Results:**

During a median (interquartile) 5.0 (2.8–5.0) years, the composite outcome occurred in 11.1% (7/63) of patients. The rate of glomerular immune deposits was 23.8% (15/63). Patients with glomerular immune deposits showed a significantly lower urine protein creatinine ratio than those without deposits (*P* = 0.033). The rate of non-responders was significantly higher in patients with glomerular immune deposits than in those without deposits (*P* = 0.033). In patients with deposits, 26.7% (4/15) developed the composite outcome, while only 6.3% (3/48) developed the composite outcome among those without deposits (*P* = 0.049). In multivariate Cox proportional hazards regression analysis, the presence of glomerular immune deposits was the only factor associated with development of the composite outcome (hazard ratio: 2.310, 95% confidence interval: 1.031–98.579, *P* = 0.047).

**Conclusion:**

Glomerular immune deposits were associated with increased risk of a composite outcome in adult MCD patients. The higher rate of non-responders in patients with deposits might be related to the poor outcome. Future study is needed.

## Introduction

Minimal change disease (MCD) accounts for approximately 10% and 30% of cases of adult primary glomerulonephritis (GN) and nephrotic syndrome, respectively [1–3]. MCD is generally thought to show excellent long-term patient and renal survival [4]. Lee et al. analyzed 1941 patients with GN. The proportion of MCD was 9.6% (187/1941), and 5.9% (11/187) of all-cause mortality was identified [2]. Chou et al. reviewed 580 patients with GN, and MCD accounted for 18.8% (109/580). Among the 109 MCD patients, dialysis dependency and all-cause mortality were identified in 0.9% (1/109) and 3.7% (4/109), respectively [5]. Because of the excellent long-term outcome, there has been little attention focused on the risk factors for poor long-term outcome in patients with MCD.

The pathogenesis of MCD remains unclear. The most accepted explanation is the presence of soluble factors, such as interleukin-13 [6], angiopoietin-like 4 [7] and CD80 [8] which initiate glomerular changes in MCD [9]. However, a smaller group shows evidence of humoral antibody or a complement response, predominantly immunoglobulin (Ig) M [10, 11] or C1q [12]. These subsets of MCD tend to be resistant to treatment [13, 14] and have poor long-term outcomes [13, 15]. Previous studies have mainly focused on clinicopathologic features of GN with mesangial IgM [15–19] or C1q deposits [14, 20–22], but other humoral factors could also play a role in the pathogenesis of MCD. Moreover, there has been no report on the clinical significance of glomerular immune deposits solely in MCD patients. Therefore, we performed the current study to determine whether glomerular immune deposits are associated with poor long-term outcome in adult patients with biopsy-proven MCD.

## Materials and Methods

### Patients

Between 2003 and 2013, 82 patients were diagnosed with biopsy-proven MCD at Seoul National University Bundang Hospital, a tertiary care hospital. A total of 63 patients were eligible for the study (Fig A in S1 Fig), after excluding those aged < 15 years (n = 4), and those with the diagnosis of focal segmental glomerulosclerosis (FSGS) in a subsequent biopsy (n = 1), inadequate specimen for immunofluorescence (IF) staining (n = 1), and less than 1 year of follow-up after the start of the study (n = 13). The study protocol complied with the Declaration of Helsinki and received full approval from the Seoul National University Bundang Hospital institutional review board (IRB number: B-1410/272-119). The need for informed consent was waived because the study did not infringe on patient privacy or health status.

### Definitions and measurements

The demographic, physiologic, laboratory and therapeutic data were gathered from the electronic medical records database. After different patient datasets were merged, verification was performed manually. The long-term outcomes analyzed were creatinine doubling, end stage renal disease (ESRD), or all-cause mortality, which was determined 5 years after the study start. ESRD development was determined from the registry database of the Korean Society of Nephrology, and all-cause mortality was determined from the database of Korean Statistics. The first date of admission for kidney biopsy was the start of our study, and the end of the study was a later date between the date of creatinine doubling, ESRD, or death. The study outcome was the composite of creatinine doubling, ESRD, or all-cause mortality.

Body mass index was calculated as weight (kg) per square of height (m^2^). Complete remission was defined as urine protein creatinine ratio (UPCR) < 0.3 g/g or < 1+ on urine dipstick for 3 consecutive measurements. Non-responders were defined as patients without complete remission throughout the treatment course. Relapse was defined as a reappearance of UPCR ≥ 3.0 g/g or ≥ 3+ on urine dipstick for 3 consecutive measurements. Serum creatinine was measured using the rate-blanked, compensated kinetic alkaline picrate Jaffe method with an automatic analyzer (Toshiba-200FR, Tokyo, Japan). The estimated glomerular filtration rate (eGFR) was calculated by using the equation of the Chronic Kidney Disease Epidemiology Collaboration [23]. Acute kidney injury was defined as a rise in serum creatinine by ≥ 26.5 μmol/L within 48 hours, or ≥ 1.5 times greater than the baseline level[24].

### Kidney pathology

For kidney pathology evaluation, all specimens were embedded in paraffin and stained with periodic acid-Schiff, Masson trichrome, methenamine silver, and hematoxylin-eosin. Glomerular lesions were defined as global sclerosis ≥ 10.0% [25], increased glomerular size or cellularity, or the presence of glomerular ischemia. Tubulointerstitial lesions were defined as non-normal reports for tubular atrophy, and interstitial inflammation and fibrosis [26]. Vascular lesions were defined as the presence of arteriolar hyalinosis and arteriosclerosis. The IF study was performed using the classic direct technique with antibodies against 5 items: IgG, IgM, IgA, C3, and C1q. Tubule, interstitium and vasculature near the evaluating glomerulus were reviewed as control tissues to exclude non-specific staining. When the reaction was appropriate, grades more than one positive were given. Pathologists evaluated the IF staining to determine whether linear, granular, peripheral, and mesangial deposits were present in the glomerulus for the 5 items which were reported semi-quantitatively as negative, trace, and 1–3 positive. We transformed the results numerically: negative to 0 point, trace to 0.5 points, and 1–3 positive to 1–3 points. Positive IF staining for the individual items was defined as above 2 points when the results of linear, granular, peripheral, and mesangial deposits in the glomerulus were summed. Glomerular immune deposits were defined when any positive IF stainings for the 5 times was present. The specific diagnosis of IgM nephropathy (IgMN) and C1q nephropathy (C1qN) were based on the following criteria: (1) intensity of IF stains scored more than two positive in IgM or C1q, or (2) one positive of intensity in IgM or C1q with evidence of mesangial changes in electron microscopic examination [i.e. electron-dense deposits (EDD), mesangial matrix expansion].

### Statistical analysis

Values were expressed as median (interquartile range, IQR) for continuous variables and percentage for categorical variables. The difference was analyzed by the Mann-Whitney U test for continuous variables and the chi-square or Fisher`s exact test for categorical variables. The Kaplan-Meier method was used for the survival curve and the statistical significance was calculated by the log-rank test. For multivariate Cox proportional hazards analysis, variables were chosen by *P* < 0.05 in univariate analysis, along with age and sex. We considered *P* < 0.05 to be statistically significant. All the analyses were performed using SPSS statistics (version 22, IBM, USA).

## Results

The median (IQR) follow-up duration for 63 patients was 5.0 (2.8–5.0) years; 49.2% (31/63) were male, and the median (IQR) age at presentation was 43.4 (27.4–65.6) years. During the follow-up period, 3 patients died from any cause (one from stroke and 2 from unidentified causes). Of 6 patients who developed creatinine doubling, 1 developed ESRD. The composite outcome was identified in 11.1% (7/63). The positive rate of glomerular immune deposits was 23.8% (15/63).

We compared baseline characteristics according to the status of glomerular immune deposits (Table 1). Although patients with deposits showed a significantly lower UPCR than those without deposits (*P* = 0.033), the level of serum creatinine and eGFR and the rate of acute kidney injury and microscopic hematuria in the positive glomerular immune deposits group were not different from those in the negative group. The rate of non-responders was significantly higher in patients with deposits than in those without deposits (*P* = 0.033).

**Table 1:** Baseline characteristics according to the status of glomerular immune deposits.

	Glomerular immune deposits		*P*
	Negative (n = 48)	Positive (n = 15)	
Age (years)	42.5 (25.3–66.9)	43.4 (32.0–62.1)	0.90
Male sex	25/48 (52.1)	6/15 (40.0)	0.41
BMI (kg/m^2^)	23.9 (22.1–26.1)	23.9 (21.3–27.1)	0.90
Systolic BP (mmHg)	116.0 (107.0–132.0)	121.0 (109.0–133.0)	0.66
Diastolic BP (mmHg)	69.5 (60.0–79.8)	73.0 (60.0–79.0)	0.77
WBC (x10^3^/μL)	6.4 (5.2–8.0)	6.4 (5.4–7.3)	0.90
Hemoglobin (g/dL)	14.2 (12.8–15.3)	14.1 (12.8–16.0)	0.57
Platelet (x10^3^/μL)	258.5 (211.0–317.3)	264.0 (186.0–292.0)	0.42
Serum glucose (mmol/L)	5.5 (5.1–6.7)	5.2 (4.8–5.6)	0.11
Total protein (g/L)	44.5 (41.0–47.8)	50.0 (41.0–56.0)	0.09
Serum albumin (g/L)	22.0 (19.3–24.0)	23.0 (20.0–29.0)	0.11
Serum creatinine (μmol/L)	73.7 (59.6–126.9)	60.7 (50.9–109.7)	0.16
eGFR (ml/min/1.73 m^2^)	89.4 (49.5–120.2)	106.8 (76.9–129.6)	0.15
Acute kidney injury	17/48 (35.4)	3/15 (20.0)	0.35
CRP (nmol/L)^a^	13.3 (1.0–28.6)	9.0 (1.0–28.8)	0.90
UPCR (g/g)	9.0 (6.3–13.3)	4.9 (3.6–9.2)	0.03
Microscopic hematuria	19/48 (39.6)	5/15 (33.3)	0.66
Serum IgG (g/L)^a^	5.4 (3.4–7.4)	7.5 (3.7–10.3)	0.17
Serum IgA (mg/L)^a^	2350.0 (1900.0–2970.0)	2240.0 (2130.0–2730.0)	0.75
Serum IgM (mg/L)^a^	1350.0 (830.0–1820.0)	1270.0 (950.0–1680.0)	0.90
Serum C3 (g/L)^a^	1.2 (1.1–1.5)	1.3 (1.1–1.5)	0.88
Serum C4 (g/L)^a^	0.3 (0.3–0.4)	0.3 (0.2–0.5)	0.85
Total steroid duration (days)	498.5 (171.8–794.0)	232.0 (159.0–839.0)	0.51
Total steroid dosage (g)	11.4 (5.8–21.4)	8.9 (6.2–15.1)	0.54
Calcineurin inhibitor use	22/48 (45.8)	5/15 (33.3)	0.39
Cyclophosphamide use	9/48 (18.8)	3/15 (20.0)	0.90
Non-responder^a^	1/48 (2.1)	3/14 (21.4)	0.03
Relapse^a^	24/46 (52.2)	6/11 (54.5)	0.89

Values were expressed as median (interquartile range) for continuous variables and n/total (%) for categorical variables. Differences were evaluated by chi square or Fisher`s exact test for categorical variables or the Mann-Whitney U test for continuous variables. BMI, body mass index; BP, blood pressure; WBC, white blood cells; eGFR, estimated glomerular filtration rate; CRP, c-reactive protein; UPCR, urine protein creatinine ratio.

^a^ represented incomplete data. The total numbers of negative/positive glomerular immune deposits of CRP, IgG, IgA, IgM, C3 and C4 were 43/14, 40/13, 43/14, 39/13, 45/14, and 45/14, respectively.

**Fig 1:**
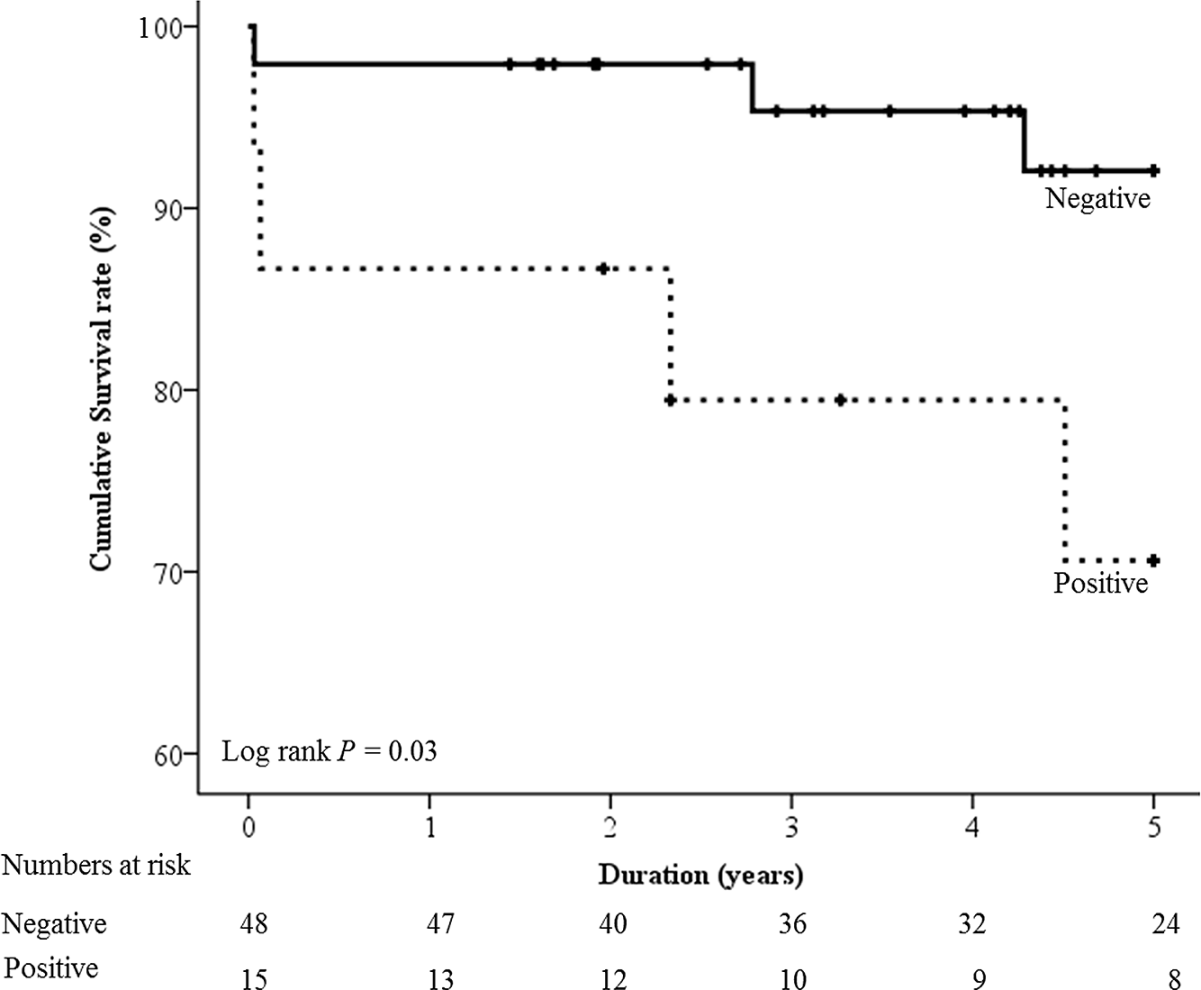
Kaplan-Meier survival curve according to the status of glomerular immune deposits. Mean (95% CI) survival of negative and positive glomerular immune deposit groups were 4.8 (4.6–5.0) years and 4.1 (3.2–5.0) years, respectively.

In patients with glomerular immune deposits, 26.7% (4/15) developed the composite outcome, while only 6.3% (3/48) of those without deposits (*P* = 0.049) developed the composite outcome. In Kaplan-Meier survival curve analysis, the positive glomerular immune deposits group showed significantly shorter survival time than the negative group (Fig 1). The glomerular immune deposits group and serum IgG level were significantly associated with the development of the composite outcome (Table 2). The confounding effects were adjusted by multivariate Cox proportional hazards regression analysis (Table 3). The presence of glomerular immune deposits was the only factor associated with the development of the composite outcome (hazard ratio [HR]: 2.310, 95% confidence interval [CI]: 1.031–98.579, *P* = 0.047).

**Table 2:** Factors associated with the composite outcome.

	Composite outcome		*P*
	Negative (n = 56)	Positive (n = 7)	
Age (years)	38.7 (26.6–65.6)	50.8 (32.0–80.5)	0.35
Male sex	29/56 (51.8)	2/7 (28.6)	0.43
BMI (kg/m^2^)	23.9 (22.1–26.7)	23.2 (21.3–27.7)	0.89
Systolic BP (mmHg)	116.5 (107.5–131.0)	133.0 (105.0–134.0)	0.46
WBC (x10^3^/μL)	6.5 (5.2–8.0)	6.1 (5.2–6.4)	0.53
Hemoglobin (g/dL)	14.2 (13.0–15.5)	14.1 (12.3–14.8)	0.50
Serum glucose (mmol/L)	5.4 (4.9–6.5)	5.3 (5.1–5.6)	0.49
Serum creatinine (μmol/L)	71.5 (59.6–110.3)	60.7 (21.5–90.1)	0.18
eGFR (ml/min/1.73 m^2^)	93.2 (51.3–120.8)	101.9 (68.9–162.8)	0.41
Acute kidney injury	16/56 (28.6)	4/7 (57.1)	0.20
CRP (nmol/L)^a^	11.4 (1.0–28.6)	13.3 (1.0–29.5)	0.78
UPCR (g/g)	8.9 (5.8–12.5)	6.2 (3.6–12.1)	0.34
Microscopic hematuria	21/56 (37.5)	3/7 (42.9)	0.90
Serum IgG (g/L)^a^	5.4 (3.4–7.5)	10.7 (6.6–12.9)	0.02
Glomerular lesions	32/56 (55.4)	4/7 (57.1)	0.90
Tubulointerstitial lesions	40/56 (71.4)	4/7 (57.1)	0.42
Vascular lesions	19/56 (33.9)	2/7 (28.6)	0.90
Glomerular immune deposits	11/56 (19.6)	4/7 (57.1)	< 0.05
Total steroid duration (days)	498.5 (161.5–831.0)	292.0 (175.0–338.0)	0.16
Total steroid dosage (g)	10.9 (5.5–21.4)	11.6 (6.9–11.8)	0.74
Calcineurin inhibitor use	25/56 (44.6)	2/7 (28.6)	0.69
Cyclophosphamide use	11/56 (19.6)	1/7 (14.3)	0.90
Non-responder^a^	2/55 (3.6)	2/7 (28.6)	0.06
Relapse^a^	28/51 (54.9)	2/6 (33.3)	0.41

Values were expressed as median (interquartile range) for continuous variables and n/total (%) for categorical variables. Differences were evaluated by Fisher`s exact test for categorical variables or the Mann-Whitney U test for continuous variables. BMI, body mass index; BP, blood pressure; WBC, white blood cells; eGFR, estimated glomerular filtration rate; CRP, c-reactive protein; UPCR, urine protein creatininie ratio

^a^ represented incomplete data. The total numbers of negative/positive composite outcomes for CRP and IgG were 50/7 and 48/5, respectively.

**Table 3:** Multivariate Cox proportional hazards regression analysis for the composite outcome.

	Beta	SE	HR	95% CI		*P*
				Lower	Upper	
Age (per 1yr increase)	0.032	0.034	1.033	0.967	1.104	0.34
Male sex (vs. female)	-0.277	0.950	0.758	0.118	4.877	0.77
Serum IgG (per 1 g/L increase)	0.128	0.146	1.137	0.854	1.513	0.38
Glomerular immune deposit (+ vs. -)	2.310	1.163	10.079	1.031	98.579	< 0.05

Age, sex and variables with *P* <0.05 in univariate analysis were integrated in the multivariate model. SE, standard error; HR, hazard ratio; CI, confidence interval.

**Fig 2:**
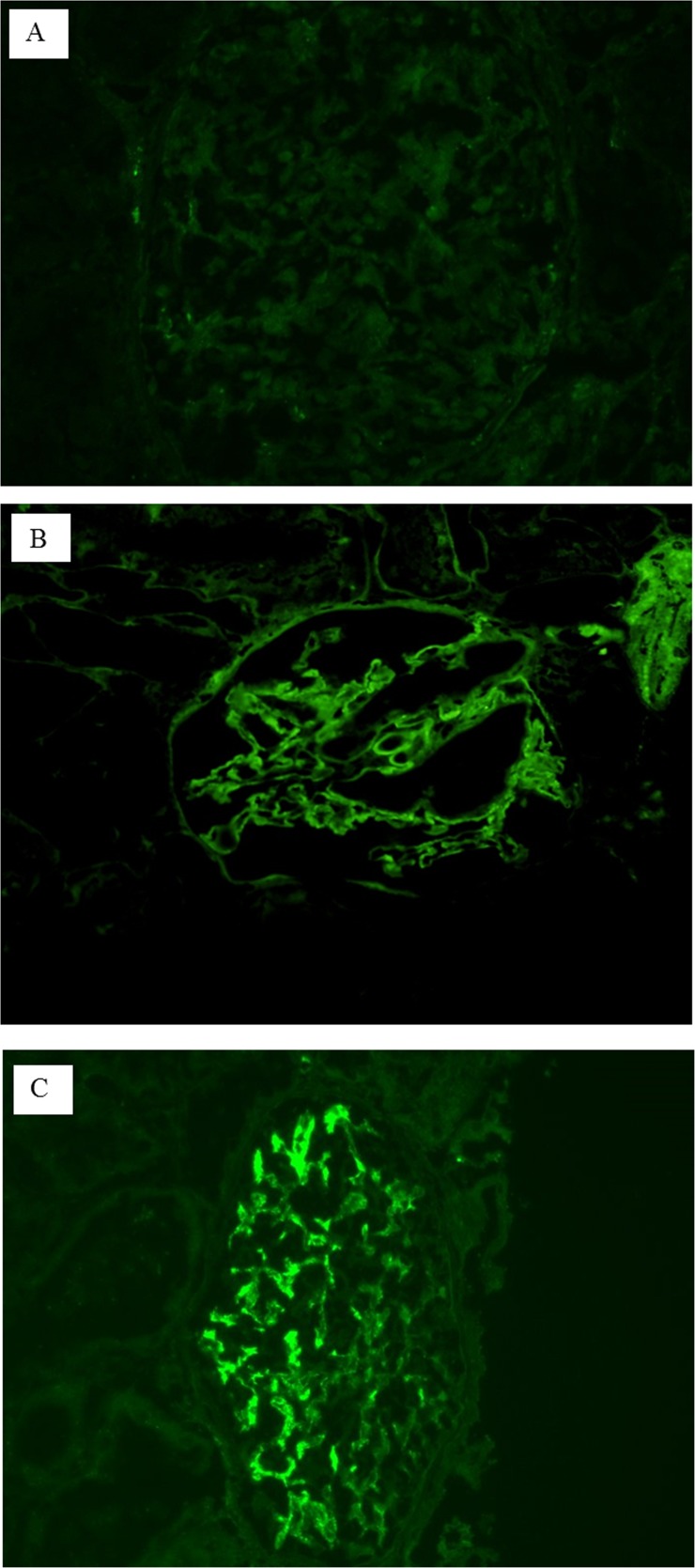
Typical results of immunofluorescence (IF) staining. A shows a negative IF result, B shows a positive result for the linear peripheral pattern and C shows a positive result for the granular mesangial pattern.

**Fig 3:**
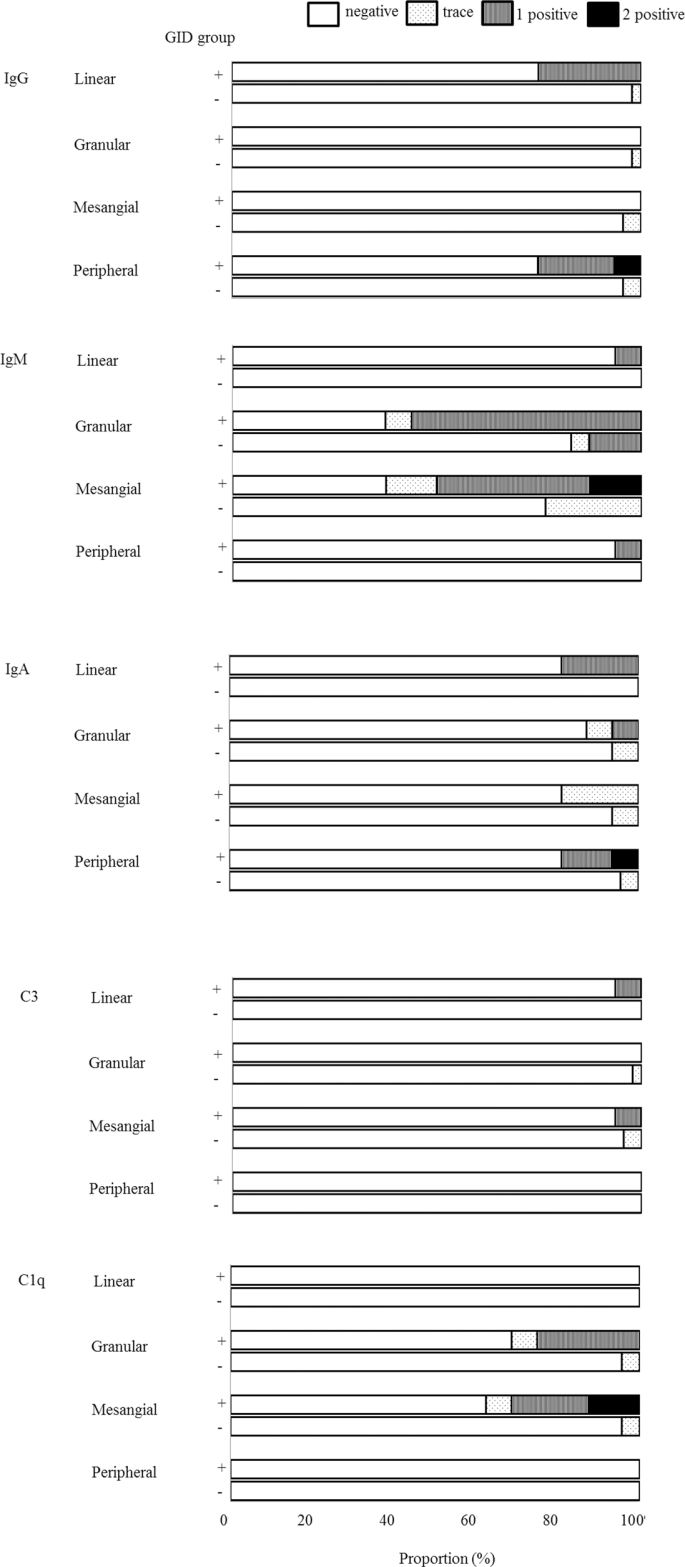
Intensity of deposits in immunofluorescence stain. GID, glomerular immune deposits.

**Fig 4:**
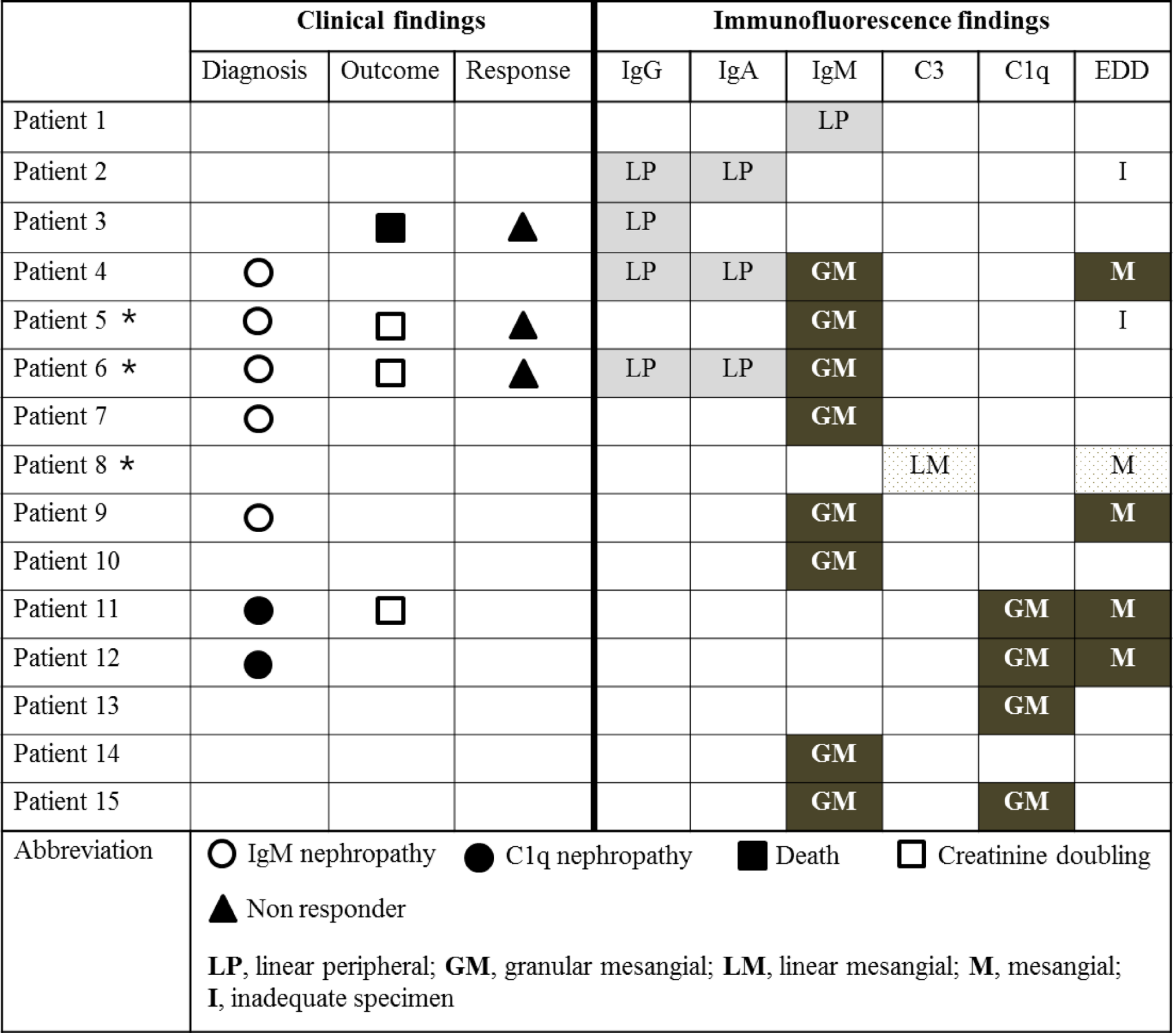
Clinical and immunofluorescence findings in patients with glomerular immune deposits. * demonstrates patients with < 3.0 g/g of urine protein creatinine ratio. EDD, electron dense deposit.

We compared pathologic characteristics according to the status of glomerular immune deposits (Table 4), and there were no differences in lesions of the glomerulus, tubulointerstitium, and vasculature. Among 15 patients in the positive glomerular immune deposits group, the most common humoral factor deposited was IgM (60%), followed by C1q (26.7%), IgG (26.7%), IgA (20.0%), and C3 (6.7%). Of these 15 patients, 5 and 2 were diagnosed with IgMN and C1qN, respectively. We demonstrated clinical and IF findings in 15 patients in the positive glomerular immune deposits group (Figs 2–4). Most IF results showed either a linear peripheral (LP) (Fig 2B) or granular mesangial (GM) pattern (Fig 2C). In the positive glomerular immune deposits group, the intensity of deposits was mostly found to be one positive result (Fig 3). Nearly all patients with IgM and C1q deposits showed a GM pattern. All patients with IgA and IgG deposits demonstrated an LP pattern. Of 8 with IgM-GM pattern deposits, 5 were diagnosed with IgMN. Of 4 with C1q-GM pattern deposits, 2 were diagnosed with C1qN. Although most of those with a composite outcome and non-responders were identified among patients with either IgM- or C1q-GM pattern deposits, 1 patient with IgG-LP pattern deposits was a non-responder and ultimately died (Fig 4).

**Table 4:** Pathologic characteristics according to the status of glomerular immune deposits.

		Glomerular immune deposits		*P*
		Negative (n = 48)	Positive (n = 15)	
Glomerular lesions		26/48 (54.2)	9/15 (60.0)	0.70
Tubulointerstitial lesions		35/48 (72.9)	9/15 (60.0)	0.34
Vascular lesions		16/48 (33.3)	5/15 (33.3)	0.90
Electron dense deposits^a^		3/43 (7.0)	5/13 (38.5)	0.01
IF stain				
	IgG	0/48 (0.0)	4/15 (26.7)	0.002
	IgA	0/48 (0.0)	3/15 (20.0)	0.011
	IgM	0/48 (0.0)	9/15 (60.0)	<0.001
	C3	0/48 (0.0)	1/15 (6.7)	0.24
	C1q	0/48 (0.0)	4/15 (26.7)	0.002
Specific diagnosis				< 0.001
	C1qN	1/48 (2.1)	2/15 (13.3)	0.14
	IgMN	0/48 (0.0)	5/15 (33.3)	< 0.001
	Typical MCD	47/48 (97.9)	8/15 (53.3)	< 0.001

Values were expressed as n/total (%). Differences were evaluated by chi square or Fisher`s exact test. IF, immunofluorescence; C1qN, C1q nephropathy; IgMN, IgM nephropathy; MCD, minimal change disease.

^a^ Among 63 patients,7 (2 in the positive group, 5 in the negative group) had inadequate specimens for electron microscopic evaluation.

## Discussion

MCD is known for excellent long-term outcomes and is considered to be the reference for the clinical features of GN [1–5]. However, poor outcomes such as ESRD and death still occur in this disease [2, 5]. A recent study by Szeto et al. showed relatively poor outcomes in 340 MCD patients: 9.4% had ESRD and 18.2% died [27]. Therefore, identification of risk factors for the development of poor outcomes is important in MCD. To our knowledge, this is the first study suggesting that glomerular immune deposits are associated with increased risk for the development of composite outcomes in adult MCD patients.

Our study showed the positive rate of glomerular immune deposits in adult MCD patients (23.8%). Consistent with previous studies [1–5], only a few patients progressed to a poor outcome. However, patients with glomerular immune deposits showed a 10 times higher risk for a poor outcome than those who did not have deposits. The reason why glomerular immune deposits are associated with poor outcome in adult MCD patients remains unclear. One possible explanation is the higher rate of non-responders in the positive deposits group than in the negative group. According to Szeto et al., treatment resistance is an independent risk factor for mortality in MCD patients (HR: 5.87, 95% CI: 1.83–18.85, *P* < 0.001) [27].

We also assume that the poor outcome found in patients positive for glomerular immune deposits is in keeping with the poor outcome shown in those with IgMN and C1qN. Over 30 years ago, several studies identified the disease entities known as IgMN and C1qN, which showed immune complex deposition in morphologically MCD patients [10–12]. According to subsequent studies, IgMN and C1qN could have a variety of phenotypes ranging from MCD to proliferative glomerulonephritis [13, 15], which suggested different disease processes from typical MCD [9]. Moreover, patients with predominantly IgM- or C1q- deposits showed poor treatment results and long-term outcomes [13–15]. To our knowledge, most previous studies on IgMN and C1qN were case series with small sample size, and only two studies directly compared IgMN or C1qN with MCD. Mubarak et al. compared 95 cases of IgMN and 267 cases of MCD in children. In their analysis, more patients with IgMN (15.7%) progressed to renal failure than those with MCD (2.5%, *P* < 0.05) [19]. However, IgMN cases in the study by Mubarak et al. had various morphologic changes: 65.9% showed glomerular mesangial proliferation, 34.1% had minor changes and 27.4% had focal segmental glomerulosclerosis, which limited direct comparison with our study results. Gunasekara et al. compared 59 MCD children with C1qN (n = 13) and without C1qN (n = 46) [22]. In their analysis, MCD patients with C1qN showed significantly shorter relapse-free periods at final follow-up, compared to MCD patients without C1qN (*P* = 0.027). However, that study did not report development of ESRD or death, and the results are not comparable with our study results.

In our study, the diagnosis of IgMN and C1qN was not consistent. The diagnosis of C1qN was made only when dominant or co-dominant deposits of C1q were accompanied by EDDs. If we used IF criteria alone [14], there were 2 more patients who could be diagnosed with C1qN in our study. The diagnosis of IgMN is a bit more confusing. Of 5 IgMN patients, 2 had evidence of EDD, but the other 3 were diagnosed solely with the results of IF staining in the absence of evidence of EDD (Fig 4: patient 5 due to 2+ intensity; patient 6–7 due to 1+ intensity with mesangial matrix expansion). We also found 3 more patients who could be diagnosed with IgMN according to IF criteria [16]. However, the inconsistency of diagnosis of IgMN and C1qN was not a problem in our study alone, since many other studies also used different criteria for the diagnosis of IgMN and C1qN [14–16, 18–21, 28]. We assume this reflects the uncertainty of IgMN and C1qN as distinct clinical disease entities [13, 15, 29].

The current study had several limitations. First, the definition of glomerular immune deposits was arbitrary, because this was the first study to expand the scope from IgM- or C1q-GM pattern deposits to any types of humoral deposits with any pattern. However, our working definition might be useful because it revealed an exceptional case of a patient who was a non-responder and ultimately died of unidentified causes, even though the diagnosis was clearly not IgMN or C1qN. The usefulness of our definition needs to be validated in a large prospective cohort. Second, the study design was retrospective. Kidney specimens or slides were not always available since many had passed the storage expiration date. Therefore, we only relied on the original pathology reports. Finally, a study from a single center and a single country limited the generalizability.

In conclusion, glomerular immune deposits were associated with increased risk of development of a composite outcome in adult MCD patients. The higher rate of non-responders among patients with glomerular immune deposits is notable, and is related to the poor outcome. Therefore, adult MCD patients with glomerular immune deposits require careful management.

## Supporting Information

S1 Fig Algorithm for the patients selection. (PPTX)
