# Supplementary material for: Glomerular Immune Deposits Are Predictive of Poor Long-Term Outcome in Patients with Adult Biopsy-Proven Minimal Change Disease: A Cohort Study in Korea
Source: PLoS One. 2016 Jan 22;11(1):e0147387. doi: 10.1371/journal.pone.0147387 (PMC4723049; doi:10.1371/journal.pone.0147387)
Supplement: S1 Fig — (PPTX) [file pone.0147387.s001.pptx]

## Slide 1
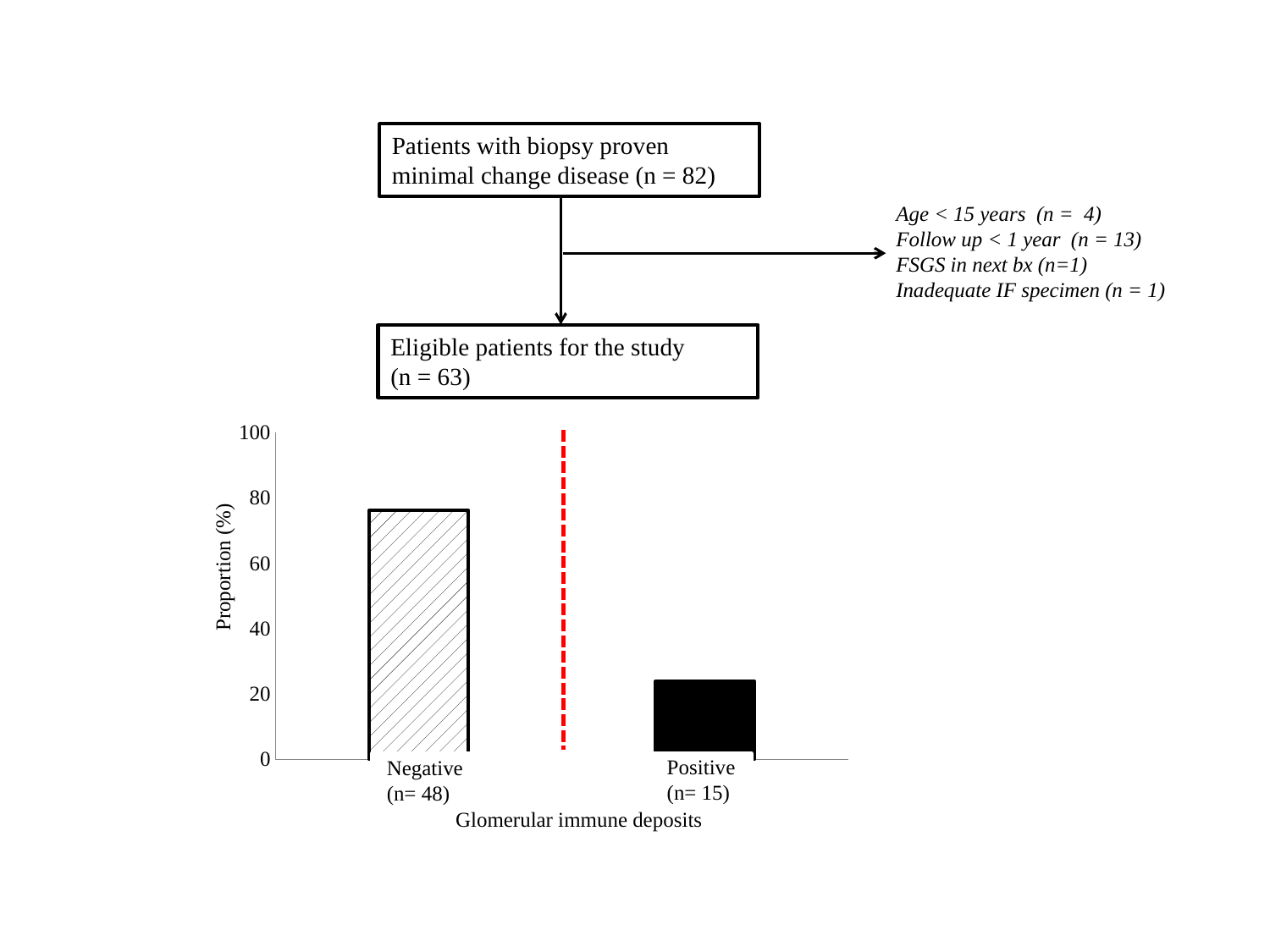

Patients with biopsy proven minimal change disease (n = 82)
Age < 15 years (n = 4)
Follow up < 1 year (n = 13)
FSGS in next bx (n=1)
Inadequate IF specimen (n = 1)
Eligible patients for the study
(n = 63)
### Chart
| Category | |
|---|---|Proportion (%)
Positive
(n= 15)
Negative
(n= 48)
Glomerular immune deposits
